# Sustainable In Silico-Supported Ultrasonic-Assisted Extraction of Oligomeric Stilbenoids from Grapevine Roots Using Natural Deep Eutectic Solvents (NADES) and Stability Study of Potential Ready-to-Use Extracts

**DOI:** 10.3390/foods13020324

**Published:** 2024-01-19

**Authors:** Mats Kiene, Malte Zaremba, Edwin Januschewski, Andreas Juadjur, Gerold Jerz, Peter Winterhalter

**Affiliations:** 1 Institute of Food Chemistry, Technische Universität Braunschweig, Schleinitzstraße 20, 38106 Braunschweig, Germany; m.kiene@tu-braunschweig.de (M.K.); g.jerz@tu-braunschweig.de (G.J.); 2 German Institute of Food Technologies, Chemical Analytics, Prof.-von-Klitzing-Straße 7, 49610 Quakenbrück, Germany; a.juadjur@dil-ev.de

**Keywords:** green solvents, natural deep eutectic solvents, grapevine roots, resveratrol, ε-viniferin, r-2-viniferin, r-viniferin, conductor-like screening model for real solvents, NADES removal process, stability study

## Abstract

Grapevine roots, as a side-stream of a vineyard, are a sustainable resource for the recovery of oligomeric stilbenoids, such as the bioactive r-viniferin. The aim of this study is to evaluate an in silico-supported method, based on the Conductor-like Screening Model for Real Solvents (COSMO-RS), for selection of environmentally friendly natural deep eutectic solvents (NADES) with regard to the extraction of grapevine roots. The most suitable NADES system for ultrasonic-assisted extraction of r-viniferin was choline chloride/1,2-propanediol. The optimal extraction parameters for r-viniferin were determined using single-factor experiments as follows: choline chloride/1,2-propanediol 1/2 mol/mol, 10 wt% H_2_O, biomass/NADES ratio 1/10 g/g, and 10 min extraction time. Under optimized conditions, the extraction yield of r-viniferin from grapevine roots reached 76% of the total r-viniferin content. Regarding stability, stilbenoids in choline chloride/1,2-propanediol remained stable during 128 days of storage at ambient temperature. However, fructose/lactic acid-based NADES were observed to degrade stilbenoids; therefore, the removal of the NADES will be of interest, with a suitable method implemented using Amberlite^®^ XAD-16N resin. As green solvents, the NADES have been used as effective and environmentally friendly extractants of stilbenoid-containing extracts from grapevine roots for potential applications in the cosmetic and pharmaceutical industry or as nutraceuticals in the food industry.

## 1. Introduction

Stilbenoids are important phytoalexins that protect plants from pathogens [1]. Moreover, stilbenoids exhibit a number of health-promoting benefits, namely antioxidant, anti-inflammatory, anticancerogenic, antiatherogenic, antiviral, and neuroprotective activities [2,3,4,5,6,7,8]. Stilbenoids are found in only a few plant families, including Vitaceae, Fabaceae, Polygonaceae, and Gnetaceae [9,10,11]. The main stilbenoids found in grapevines are *trans*-resveratrol, *trans*-ε-viniferin, r-2-viniferin (vitisin A), and r-viniferin (vitisin B). Bioactive properties indicate these as potential additives in dietary supplements or cosmetics. Promising natural sources for stilbenoids are side-streams of wine production. In particular, the lignified stem tissue and rootstocks of grapevine contain higher amounts of stilbenoids [12,13,14]. In cases where vineyards need to be cleared for any reason (e.g., old age or new resistant planting of cultivars), grapevine roots provide a sustainable source of oligomeric stilbenoids (e.g., r-2-viniferin and r-viniferin) [1,15]. Stilbenoids are considered to be responsible agents in the protection against wood rotting [1]. Grapevine cane and root extracts have shown strong tyrosinase inhibitory effects. For this reason, they are used to treat skin diseases related to hyperpigmentation, and they can also serve as skin-lightening agents. In addition, skin care as well as photoprotective effects have been reported for stilbenoid-containing extracts [16,17,18,19,20]. Due to these bioactivities, extracts from grapevine side-streams are of interest for cosmetic formulations with specific functionalities. The exploitation of these side-streams of wine production through sustainable extraction techniques using novel, solvent-free, ecologically safe, and environmentally friendly extractants as replacements for conventional organic solvents is, therefore, of scientific and economic interest.

A class of green solvents—whose properties, such as low flammability, low vapor pressure, stability, lack of toxicity, biodegradability, and lower costs, correspond to the key topics of Green Chemistry (e.g., Topic 5: safer solvents)—are the natural deep eutectic solvents (NADES) [21,22,23,24]. The NADES are a specific group of deep eutectic solvents (DES) introduced by Abbott et al. (2003) [25]. The DES are defined as liquid mixtures of pure compounds whose melting point temperatures are below those of the individual components and deviate strongly from the ideal liquid mixture [26,27]. The melting point depressions occur due to strong intermolecular, non-covalent interactions, primarily hydrogen bonding [28]. These effects result in the formation of a supramolecular lattice that is energetically favored. Water can also be incorporated into this lattice up to a specific level [23]. The term natural deep eutectic solvent was introduced by Choi et al. (2011) [29]. The NADES are composed of components such as carbohydrates, organic acids, or amino acids, which are present in large quantities in the primary metabolism of plants. In general, the NADES are formed by combining hydrogen bond acceptors (HBAs) and hydrogen bond donors (HBDs). Amino acids or quaternary ammonium salts are used as HBAs, and organic acids or carbohydrates act as HBDs. Natural substances with alcohol, carboxyl, keto, and amino groups act simultaneously as HBAs and HBDs.

This huge number of possible compositions of HBAs and HBDs, which may be used in different molar ratios as well as different water contents, results in a wide variety of NADES systems that can be prepared. In order to minimize the number of preliminary tests, which is in line with the concept of Green Chemistry, the Conductor-like Screening Model for Real Solvents (COSMO-RS), developed by Klamt and co-workers, is used to select the most suitable combinations of HBAs and HBDs to form optimized NADES systems for the extraction of stilbenoids from grapevine roots [30,31,32]. For this purpose, a quantum chemical calculation of the charge density distribution on the surface of the NADES mixtures and the stilbenoids was carried out using the COSMO-RS (cf. Supplementary Material Figures S1–S4). In the next step, these results were used to calculate the chemical potentials of the stilbenoids in the NADES mixtures. The chemical potentials resulting from these calculations are the basis for further thermodynamic equilibrium properties, such as the activity coefficient. One significant benefit is that the activity coefficients of the stilbenoids in the NADES mixtures can be calculated using the COSMO-RS without the need for previously measured experimental data. In a screening, the calculated activity coefficient was used to show the solubility trend of the stilbenoids in the NADES mixtures, where a lower activity coefficient indicates a better solubility of the stilbenoids in the respective NADES systems. In previous studies, the COSMO-RS approach had been used to select suitable NADES to extract polyphenols from various plant materials [33,34,35].

The NADES systems offer many advantages as a novel extractant. The polarity and viscosity of the NADES can be adjusted through the selection of starting compounds, the molar HBA/HBD ratio, and the water content. This tenability enables tailored NADES systems depending on the target compounds and the sample material to be extracted, leading to a wide range of potential applications, such as nutraceuticals in the food industry [36]. Finally, extracts could be used after the removal of the NADES, e.g., by using XAD resins with food-grade quality [37]. Ready-to-use extracts still containing NADES could also be used, which is particularly interesting for cosmetic formulations [38,39,40]. The advantage of ready-to-use extracts is that the extraction agents do not need to be removed prior to use in the final product. Previous research has also shown that NADES can increase the stability of polyphenols [41,42]. Especially in cosmetic formulations, NADES components could induce positive effects, such as the skin-lightening effect of betaine [43]. Resveratrol containing NADES extracts with potential use in cosmetic formulations was already described using materials from *Polygonum cuspidatum* or *Gnetum gnemon* [10,11]. The successful extraction of resveratrol and ε-viniferin from grapevine canes of different *Vitis* species [35,44,45] demonstrates the potential of the NADES as an extractant for stilbenoids, although there is still a scientific gap in the NADES extraction of tetrameric stilbenoids. Furthermore, the storage stability of the NADES extracts containing oligomeric stilbenoids, which could be used as ready-to-use extracts in cosmetic formulations, has not been studied.

Therefore, the main aim of this study was to use the COSMO-RS to select potential NADES systems for obtaining bioactive stilbenoids from grapevine roots (*Vitis vinifera* cv. “Gewürztraminer” and “Selection Oppenheim 4” as rootstock). To the best of our knowledge, simultaneous NADES extraction of *trans*-resveratrol, ε-viniferin, r-2-viniferin, and r-viniferin from grapevine roots has not been previously investigated. The study examined the correlation between solubility trends calculated through the COSMO-RS and the experimental extraction rates measured using UHPLC-UV after ultrasonic-assisted extraction. Using the most suitable NADES, its water content (wt%), HBA/HBD molar ratio (mol/mol), biomass/NADES ratio (b/N ratio, grams of dried grapevine root powder per gram of NADES), and extraction time (min) were optimized using single-factor experiments to maximize the extraction content of stilbenoids. Stability over the storage time of the stilbenoids in ready-to-use NADES extracts was evaluated through a comparison of the NADES grapevine root extract and NADES resveratrol solutions. In addition, the effectiveness of the XAD-16N resin material was tested for the removal of stilbenoids from NADES mixtures.

## 2. Materials and Methods

### 2.1. Chemicals

Double-deionized water (Nanopure^®^, Werner GmbH, Leverkusen, Germany) was used. Ethanol (HPLC grade) and acetic acid (LC-MS grade) were purchased from Fisher Scientific (Loughborough, UK). Acetonitrile (HPLC and LC-MS grade) was obtained from Honeywell Specialty Chemicals (Seelze, Germany). Formic acid (HPLC grade) and lactic acid (90%) were purchased from VWR Int. S.A.S (Darmstadt, Germany). For NADES preparations, betaine (98%) and urea (99.5%) were purchased from Fisher Scientific (Loughborough, UK). D-Fructose (99%) and D-glucose (99%) were obtained from Carl Roth (Karlsruhe, Germany). Amberlite^®^XAD-16N, choline chloride (>98%), and 1,2-propanediol (99%) were purchased from Sigma-Aldrich (Deisenhofen, Germany). *Trans*-resveratrol standard (>98%) was obtained from Carl Roth (Karlsruhe, Germany), and *trans*-ε-viniferin (>94%, *λ* 280 nm, in-house method, [35]) was isolated from the commercial product Vineatrol^®^30 (Breko GmbH, Bremen, Germany). Stilbenoid tetramers r-2-viniferin (95%, *λ* 280 nm) and r-viniferin (91%, *λ* 280 nm) were isolated from de-colored Vitisin^®^ Powder (Actichem, Montauban, France) using an in-house method [46]. Each sample for UHPLC and LC-MS analysis was filtered through 0.2 µm PTFE syringe filters from Agilent Technologies (Waldbronn, Germany).

### 2.2. Plant Material

Grapevine roots (“Selection Oppenheim 4”, *Vitis berlandieri* × *Vitis riparia*, nobel vine “Gewürztraminer Klon 46-106”, collected in Würzburg, Germany) were lyophilized until a constant weight was reached (freeze dryer Christ Alpha 2–4, Osterode, Germany). The samples were ground with a cutting mill (parallel section rotor, bottom sieve with trapezoid holes of 2 mm, Retsch SM 1, Haan, Germany).

### 2.3. Computational Calculation of Compound Specific Activity Coefficients

A previous study evaluated the suitability of HBA and HBD combinations as potential NADES extractants for *trans*-resveratrol and ε-viniferin [35]. A software-based calculation applying the Conductor-like Screening Model for Real Solvents (COSMO-RS) was used to calculate the activity coefficient (*γ*) of *trans*-resveratrol and ε-viniferin in different NADES systems. Calculations of the activity coefficients for the stilbene oligomers r-2-viniferin and r-viniferin were performed based on the same model. The activity coefficient $\gamma_{i}^{\infty ,s}$ of solute *i* infinitely diluted in solvent *s* is defined as (Equation (1)):

(1) $$\gamma_{i}^{\infty ,s}=\exp\;\left(\left(\mu_{i}^{s}\;-{\;\mu }_{i}\right)/\mathrm{RT}\right)$$

where $\mu_{i}^{s}$ is the chemical potential of the solute *i* in the solvent *s* and $\mu_{i}$ is the chemical potential of pure solute *i*. As the activity coefficient decreases, the tested HBA and HBD combination is postulated to be better able to solubilize and, therefore, to extract *trans*-resveratrol, ε-viniferin, r-2-viniferin, and r-viniferin, respectively.

In the initial stage, the chemical structures of *trans*-resveratrol, ε-viniferin, r-2-viniferin, r-viniferin, choline chloride, and betaine were retrieved from ChemSpider [47] as SMILES and entered into the software BIOVIA COSMOconfX (version 22.0.0, Dassault Systèmes, Vélizy-Villacoublay, France). The conformers of *trans*-resveratrol, ε-viniferin, r-2-viniferin, r-viniferin, choline chloride, and betaine were calculated in COSMOconfX using the Becke-Perdew functional (BP), a triple-zeta valence polarization with diffuse functions (TZVPD) and a fine grid marching tetrahedron cavity (FINE) template. The template includes a full geometry optimization with the density functional theory (DFT) at the BP-TZVP level, with a consecutive BP-def2-TZVPD single-point calculation and a FINE cavity for the COSMO calculation. The conformers were considered as a Boltzmann-weighted mixture of conformers for the calculations. The maximum number of conformers from the geocheck clustering was set at 40, and 75 was the maximum number of conformers. Calculated structures were verified to be true minima using vibrational frequency analysis. All other compounds were taken from the database BIOVIA COSMObaseEditor (version 22.0.0, Dassault Systèmes).

For modeling the HBA choline chloride, the so-called ion pair approach was used. Thereby, the COSMO-RS optimized structure of choline chloride could be described as a single non-dissociated molecule [48]. The organic acids were treated as protonated compounds. NADES were treated as binary mixtures of HBDs and HBAs at different stoichiometric ratios (3/1, 2/1, 1/1, 1/2, and 1/3) within the framework of COSMO-RS. For this approach, only the conformer with the lowest energy level of *trans*-resveratrol, ε-viniferin, r-2-viniferin, and r-viniferin was used. Additionally, only the conformers of HBAs and HBDs with the lowest energy level were included to allow a screening with around 1200 potentially suitable NADES systems in a reasonable amount of time. Therefore, the software BIOVIA COSMOthermX (Version 22.0.0, Dassault Systèmes) [49] with the BP_TZVPD_FINE_22.ctd parameterization was used to calculate the activity coefficients of *trans*-resveratrol, ε-viniferin, r-2-viniferin, and r-viniferin in NADES at 25 °C, and infinite dilution with 0–70 wt% of water.

### 2.4. Preparation of Natural Deep Eutectic Solvents

NADES were prepared using the heating method according to Dai et al. (2013) [23]. The mixtures of HBAs and HBDs, shown in Table 1, were heated at 70–80 °C with constant stirring until a homogeneous, clear liquid was obtained.

### 2.5. Ultrasonic-Assisted NADES-Extraction of Stilbenoids

The extraction procedure was performed according to Kiene and co-workers with slight modifications [35]. Approximately 200 mg of the lyophilized and ground grapevine roots were placed in centrifuge tubes (20 mL), mixed with 2 g of NADES or organic extraction solvents, and mixed with a vortex mixer. Subsequently, the solutions were extracted using an ultrasonic homogenizer equipped with a 1/8” ultrasonic horn (Branson Ultrasonics Sonifier S450A, Danbury, CT, USA) with an output control of 20% for 4.5 min. The extract was centrifuged at 8000 rpm for 5 min. After centrifugation, the supernatant was filtered using a fiberglass membrane disc filter (1 µm pore size, 25 mm i.d., Chromafil Xtra GF-100/25, Macherey & Nagel, Düren, Germany). The filtered solution was then transferred to volumetric flasks and topped up to a defined volume with water/acetonitrile (1/1; *v*/*v*). The extraction was performed in triplicates for the grapevine root powder.

For determination of the extraction content, a calibration curve for *trans*-resveratrol, *trans*-ε-viniferin, r-2-viniferin, and r-viniferin was prepared (cf. Supplementary Material Table S5). The extraction contents of stilbenoids in grapevine roots were measured using UHPLC-UV (cf. Section 2.10) and reported as milligrams per gram of dry weight (DW).

### 2.6. Optimization of the Extraction Process

The extraction process optimization was performed using single-factor experiments, with parameters optimized successively.

#### 2.6.1. Influence of Water Content in NADES

To analyze the effects of varied water contents NADES systems, extractions were performed using Ch/Pdiol 1/2 mol/mol with five different water contents (10, 20, 30, 40, and 50 wt%). The extraction process was carried out according to the general method (cf. Section 2.5). Each experiment was performed in triplicate.

#### 2.6.2. Influence of HBA/HBD Molar Ratio

Extractions for determining the effect of HBA/HBD molar ratio were performed using Ch/Pdiol, 10 wt% H_2_O with six various molar ratios (1/1, 1/2, 1/3, 1/4, 1/5, and 1/6 mol/mol). The extraction process was carried out according to the general method (cf. Section 2.5). Each experiment was performed in triplicate.

#### 2.6.3. Influence of the Biomass/NADES Ratio

To determine the effect of biomass/NADES ratio, extractions were performed using Ch/Pdiol 1/2 mol/mol, 10 wt% H_2_O with four different biomass/NADES ratios (1/5, 1/10, 1/20, and 1/30 g/g). The extraction process was carried out according to the general method (cf. Section 2.5). Each experiment was performed in triplicate.

#### 2.6.4. Influence of the Extraction Time

Extractions were performed using Ch/Pdiol 1/2 mol/mol, 10 wt% H_2_O (1/10 g/g b/N ratio). To evaluate the effect of time on the stilbenoid extraction contents, extractions were carried out for 2, 4.5, 7, 10, 12.5, and 15 min. The extraction process was carried out according to the general method (cf. Section 2.5). Each experiment was performed in triplicate.

### 2.7. Successive Ultrasonic-Assisted Extraction

To determine the total extraction yield of the stilbenoids, a successive extraction (four times in total) based on the procedure of Ewald et al. (2017) was performed [14]. Approximately 2.5 g of grapevine roots were weighed into a centrifuge tube (50 mL). After adding 20 mL of an ethanol/water mixture (80/20; *v*/*v*), the ultrasonic-assisted extraction was carried out for 4.5 min (cf. Section 2.5). A centrifugation step (8000 rpm for 5 min, Hettich Universal 30F, Lauenau, Germany) was performed after extraction. Supernatants were combined and topped up to 100 mL. The resulting extract was dried under nitrogen and then reconstituted with an ethanol/water mixture (80/20; *v*/*v*) to obtain a 10-fold concentrated solution.

### 2.8. Stability Studies

#### 2.8.1. Stability Study of Stilbenoids in Choline Chloride/1,2-Propanediol NADES

Solutions of 100 mg/g grapevine roots in Ch/Pdiol 1/2, 10 wt% H_2_O were prepared as described in Section 2.5 and were used for the longer-term stability study. Measurements of the pH value were performed using an MP 225 pH meter (Mettler-Toledo, Gießen, Germany). The vials were stored protected from light at ambient temperature (22 °C), and stilbenoid contents were measured after 0, 36, 69, 93, and 128 days using UHPLC-UV. All experiments were performed in duplicate, with one analytical duplicate in each case.

#### 2.8.2. Stability Study of Stilbenoids in Fructose/Lactic Acid NADES

Solutions of 2.08 mg/g resveratrol reference standard and 10 mg/g grapevine roots in Fru/LA 1/2, 20 wt% H_2_O were prepared as described in Section 2.5 and were used for the longer-term stability study. Measurements of the pH value were performed using an MP 225 pH meter (Mettler-Toledo, Gießen, Germany). The vials were stored at ambient temperature (22 °C) in the dark, and stilbenoid contents were measured after 1, 2, 3, 7, 10, 17, 24, 45, and 59 days using UHPLC-UV. All experiments were performed in duplicate, with one analytical duplicate in each case.

### 2.9. NADES Removal on Amberlite^®^ XAD-16N Resin

The NADES system Ch/Pdiol 1/2; 10 wt% H_2_O was used for the preparation of a grapevine root NADES extract (cf. Section 2.5). Prior to the removal of NADES, the extract’s stilbenoid content was determined using UHPLC-UV to identify its yield (cf. Section 2.10). An aliquot of the NADES extract was subjected to Amberlite^®^ XAD-16N macroporous resin chromatography column to remove the stilbenoids from the NADES system. After adsorption, the column was washed with 400 mL of double-deionized water, and the stilbenoids were eluted with 250 mL of ethanol. The ethanolic eluent was dried using lyophilization (freeze dryer Christ Alpha 2–4, Osterode, Germany). Stilbenoid contents in the NADES-free extract were measured using UHPLC-UV (cf. Section 2.10).

### 2.10. UHPLC-UV Analysis

For quantification of stilbenoids, a UHPLC system from Agilent Technologies (Waldbronn, Germany), with a high-speed pump (1290 Infinity II series), multicolumn thermostat (1290 Infinity II series), vialsampler (1290 Infinity II series), a diode array detector (1290 Infinity II series), and Open Lab CDS 3.4 (Agilent Technologies, Waldbronn, Germany) was used. The separation was achieved on a Zorbax Eclipse Plus C18 column (1.8 µm, 50 × 2.1 mm, Agilent Technologies, Waldbronn, Germany) according to Kiene and co-workers [35]. For this, the mobile phase consisted of water/acetonitrile/formic acid (100/10/0.1; *v*/*v*/*v*) (A) and acetonitrile (B). The separation was carried out at 60 °C with a flow rate of 0.4 mL/min, under the following conditions: 0 min (0% B), 1.73 min (0% B), 2.73 min (16% B), 6.2 min (45% B), 6.4 min (0% B), and 7.2 min (0% B). *Trans*-resveratrol was quantified at *λ* 306 nm and *trans*-ε-viniferin, r-2-viniferin, and r-viniferin were quantified at 324 nm, respectively. The quantification parameters of the UHPLC-UV methodology are listed in Supplementary Material Table S5.

### 2.11. HPLC-UV-MS/MS Analyses

For qualification and peak identification purposes, an HPLC-ESI-MS system consisting of a binary HPLC pump (1100 series) and autosampler (1200 series) from Agilent Technologies (Waldbronn, Germany) equipped with an LC-ESI-MS/MS ion-trap system (HCT Ultra ETD II, Bruker Daltonics, Bremen, Germany) was used. Mass spectra were recorded in the negative ionization mode with the capillary voltage set at 3500 V, end plate −500 V, and capillary exit −115.0 V. Drying gas was nitrogen at 330 °C, and 10.0 L/min flow rate with nebulizer pressure of 50 psi, target mass setting *m*/*z* 350, scan range from *m*/*z* 100–2000 in Ultra Scan mode, fragmentation amplitude 1 V. Compass Hystar Software (version V. 3.2, Bruker Daltonics) was used for analysis and data collection. HPLC separation was adapted from Kiene and co-workers and performed on a C18-column (Aqua 3u, 100 Å, 3 μm, 150 mm × 2.0 mm i.d.) from Phenomenex (Aschaffenburg, Germany) with a guard column of the same material at a flow rate of 0.20 mL/min [35]. The mobile phase consisted of 2% aqueous acetic acid (*v*/*v*) (A) and acetonitrile (B). HPLC conditions for ESI-MS/MS analysis were 0 min (20% B), 20 min (30% B), 40 min (50% B), 50 min (80% B), 55 min (80% B), 60 min (20% B), 70 min (20% B).

### 2.12. Statistical Analysis

Experimental results were obtained as the mean value ± standard deviation (SD) (*n* = 3). Statistical analyses were performed using the software OriginPro 2022 (OriginLab Corporation, Northampton, MA, USA), version 9.9.0.225. The significance of difference was assessed using ANOVA followed by Tukey’s HSD test. Differences were considered significant when the *p* value was <0.05 and were designated by lower-case letters.

## 3. Results and Discussion

**Figure 1:**
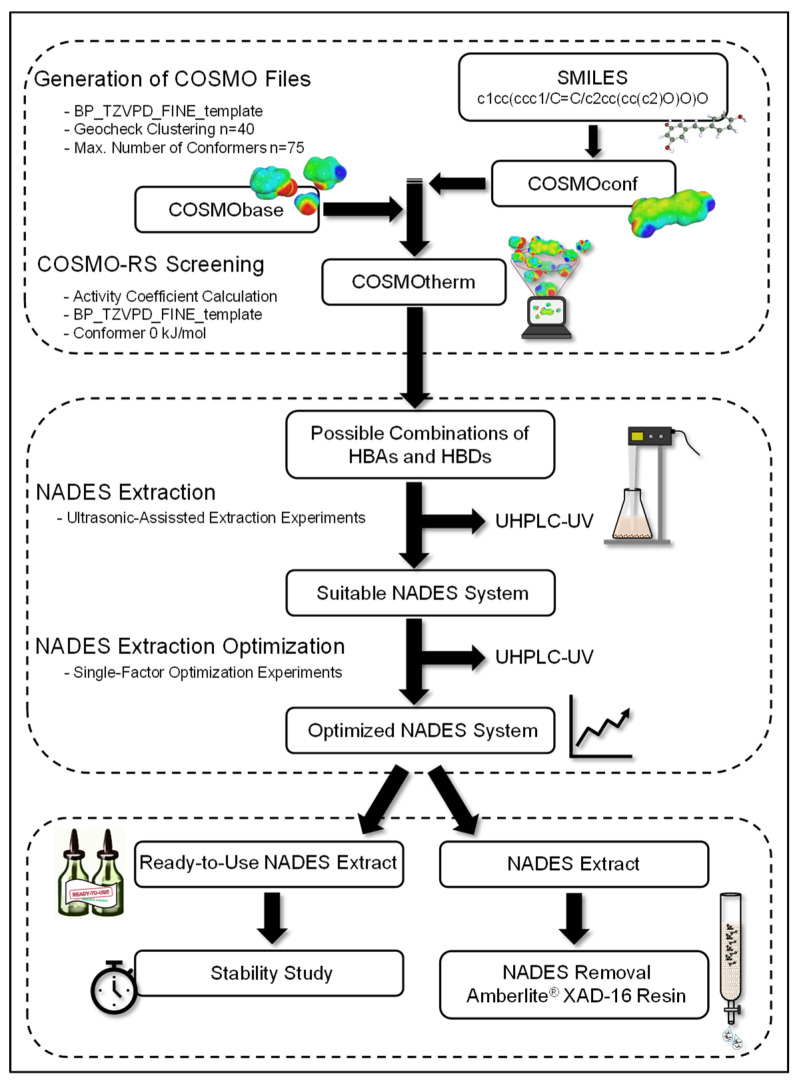
Experimental setup including the three main steps for in silico-supported selection of suitable NADES, NADES extraction experiments, and NADES applications applied in this work.

The environmentally friendly NADES are promising extractants for bioactive stilbenoids. They could be prepared from different combinations of HBAs and HBDs, which have a huge influence on the physicochemical properties of the NADES. These properties, such as polarity or solution capacity, influence their extraction efficiency. Figure 1 shows the experimental setup used for the extraction of grapevine roots (*Vitis vinifera* cv. “Gewürztraminer” and “Selection Oppenheim 4” as rootstock) with the NADES.

In the first step, the in silico-supported calculation for the selection of suitable NADES was carried out. The following steps involved the selection of promising NADES systems for ultrasonic-assisted extraction experiments of stilbenoids, whose extraction contents were measured using UHPLC-UV. The water content, HBA/HBD molar ratio, biomass/NADES ratio, and extraction time were optimized for the most suitable NADES system through single-factor experiments to maximize the extraction content of resveratrol, ε-viniferin, r-2-viniferin, and r-viniferin. Finally, the shelf life of ready-to-use NADES extracts was evaluated, and a method for recovering stilbenoids from the NADES with the Amberlite^®^ XAD-16N resin was established.

### 3.1. In Silico-Supported Selection of HBAs and HBDs for Stilbenoid Extraction

**Figure 2:**
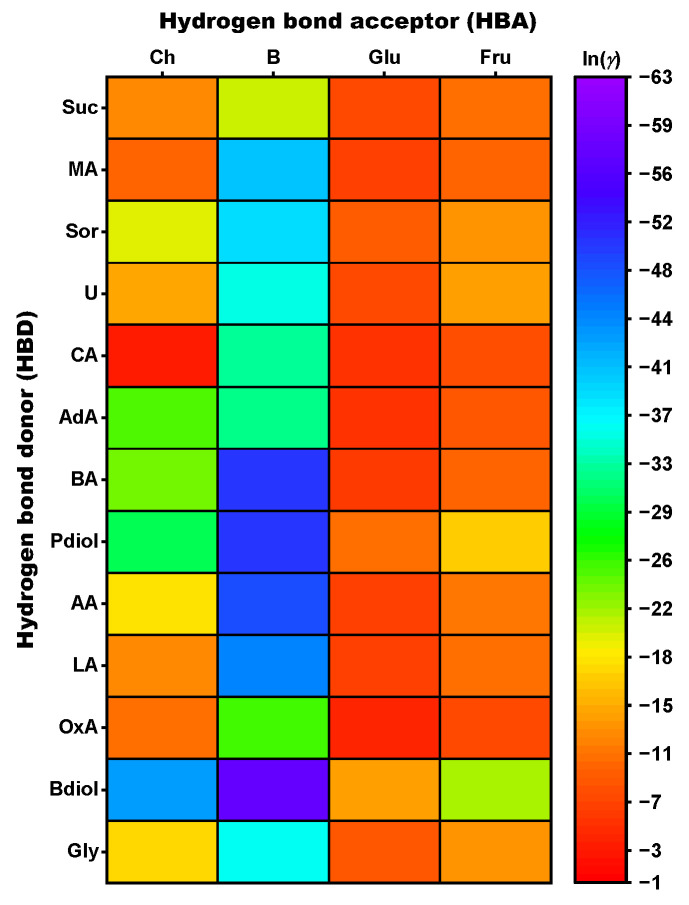
Heat map of the activity coefficients ln *γ* calculated with COSMO-RS (25 °C) of r-viniferin in different NADES. The molar ratio is 1/1, and the water content for all NADES is 0 wt%. Abbreviations: AA, acetic acid; AdA, adipic acid; BA, benzoic acid; B, betaine; Bdiol, 1,4-butanediol; Ch, choline chloride; CA, citric acid; Fru, fructose; Glu, glucose; Gly, glycerol; LA, lactic acid; MA, malic acid; OxA, oxalic acid; Pdiol, 1,2-propanediol; Sor, sorbitol; Suc, sucrose; and U, urea.

In the present study, 52 NADES with four different HBAs (choline chloride (Ch), betaine (B), glucose (Glu), and fructose (Fru)) and 13 different HBDs were preselected through in silico-screening based on the calculated activity coefficients of resveratrol, ε-viniferin, r-2-viniferin, and r-viniferin in the NADES systems. This screening was used to select NADES systems able to extract these stilbenoids from grapevine roots. Choline chloride and betaine-based NADES systems were selected due to their comprehensive description in the literature and their potential application in cosmetic formulations [10,11,38]. Glucose and fructose-based NADES systems could be used for applications in the food industry. Various polyols, organic acids, sugar-based sweeteners, and urea (U) were selected as HBDs. The suitability of HBA and HBD combinations as potential NADES extractants for the four stilbenoids was calculated using the COSMO-RS implemented activity coefficient *γ*. COSMOtherm reports the activity coefficient as ln *γ*, and as this value decreases, the combination of HBAs and HBDs is better able to solubilize and extract resveratrol, ε-viniferin, r-2-viniferin, and r-viniferin, respectively [34]. In these COSMOtherm calculations, HBAs and HBDs are included as solids to simplify the calculation, whereby it is assumed that the mixture of both substances in a defined molar ratio and a specific water content forms a supramolecular liquid that dissolves the target compounds. Calculations were performed with different molar ratios (3/1, 2/1, 1/1, 1/2, and 1/3) and water contents (0–70 wt%) for each combination of HBAs and HBDs, resulting in a total of 1200 NADES systems. The heat map in Figure 2 shows the solubility trend of r-viniferin in a subset of these different combinations (molar ratio 1/1, water content 0 wt%), showing that the betaine-based NADES gave higher solubilities of r-viniferin than systems with choline chloride, glucose or fructose. Based on this screening, 1,4-butanediol (Bdiol) with ln *γ* = −57.4, 1,2-propanediol (Pdiol) with ln *γ* = −50.3, and benzoic acid (BA) with ln *γ* = −50.1 were promising HBDs for the preparation of suitable NADES with betaine as HBAs. These combinations should be the most suitable for solubilizing r-viniferin. When these donors were used with choline chloride as HBAs, the activity coefficients of r-viniferin increased slightly for their respective donors (HBDs: 1,4-butanediol ln *γ* = −42.8, 1,2-propanediol ln *γ* = −30.1, and adipic acid (AdA) ln *γ* = −25.0). Using glucose and fructose as HBAs failed to achieve good solubility results for r-viniferin in any combination tested. We note that 1,4-butanediol (ln *γ* = −21.7) and 1,2-propanediol (ln *γ* = −16.4) would be the most useful HBDs when fructose is used as HBAs. In combination with glucose, both HBDs have a lower calculated solubility (Glu/Bdiol ln *γ* = −13.4, Glu/Pdiol ln *γ* = −10.6).

The calculated activity coefficients of resveratrol, ε-viniferin, and r-2-viniferin in the NADES systems followed a similar trend as r-viniferin. However, the calculated activity coefficients decreased with an increasing degree of oligomerization (Supplementary Material Figure S5 and Tables S1–S4). Another trend observed for the four stilbenoids was a decrease in the calculated solubility efficiency of the NADES with increasing water contents. The similarly calculated activity coefficients of the stilbenoids resulted in their sigma profiles not differing significantly. Overall, the COSMO surface area increases from monomer to tetramer, resulting in much lower calculated activity coefficients for the tetramers in the same NADES system. All stilbenoids have a large surface area in the apolar region between ±0.01 e/Å^2^, with two maxima at −0.007 and 0.005 e/Å^2^, indicating a hydrophobic character, while the surface area of r-viniferin in the range between −0.009 and −0.002 e/Å^2^ was slightly larger than that of r-2-viniferin. The maxima in the polar region at −0.018 e/Å^2^ and 0.012 e/Å^2^ indicate the property of inducing hydrogen bonds.

During the NADES preparation, it was observed that the NADES could only be prepared from choline chloride or betaine with the most suitable HBD 1,4-butanediol by adding 30 wt% H_2_O (ln *γ* = −22.1 r-viniferin). Instead, 1,2-propanediol was used to produce the NADES with a lower water content (20 wt%), which, according to the COSMO-RS calculations (ln *γ* = −26.1 r-viniferin), improves the solubility of the stilbenoids. Furthermore, the NADES for potential use in food products consisting of fructose and lactic acid (LA) was chosen as the medium-quality NADES. Also, Glu/U 3/1, 60 wt% H_2_O, was selected as unsuitable NADES for the extraction of stilbenoids. Subsequent extraction experiments and evaluations of the calculated solubility trend include the NADES with four different HBAs, three different HBDs, and water contents in the range of 10–60 wt%. The COSMO-RS-based screening was used to preselect potentially suitable HBAs and HBDs, which are widely available, cheap, renewable, and biodegradable. After evaluating their extraction properties, the other NADES properties, such as molar ratio and water content, were optimized for the most suitable system (cf. Section 3.3). However, the influence of the water content and the molar ratio on the viscosity of the NADES and, thus, on the mass transfer, which is important for the extraction efficiency, could only be determined experimentally.

The correlation between the COSMO-RS calculated ln *γ* values and experimental extraction contents of stilbenoids in various NADES (determined using UHPLC-UV), plotted in Supplementary Material Figure S6, was evaluated using Pearson’s coefficient *r* (α = 0.05). The negative correlation between ln *γ* and the measured extraction levels of the four stilbenoids means that an *r* value less than −0.5 indicates a high degree of correlation, while 0 indicates no relationship. Based on the ln *γ* values calculated with the COSMO-RS and the experimentally determined values, the extractive suitability of the NADES systems shows the following decreasing trend: Ch/Pdiol 1/2 > B/Pdiol 1/1 > B/Pdiol 3/1 > Fru/LA 1/2 > Glu/U 3/1. For resveratrol, the correlation coefficient was −0.97, for ε-viniferin −0.98, for r-2-viniferin −0.83, and for r-viniferin −0.83. The extractability of various NADES has been calculated using the COSMO-RS model. However, it should be noted that the trend of the extractability of the NADES of resveratrol and viniferin could be reproduced more reliably with the in silico model than for the two tetramers. Furthermore, this COSMO-RS-supported workflow was demonstrated to be capable of generating suitable NADES from a variety of combinations of HBAs and HBDs for the extraction of resveratrol, ε viniferin, r-2-viniferin, and r-viniferin from grapevine roots. This successful in silico-selection made it possible to minimize the number of preliminary tests, in line with the concept of Green Chemistry [24].

### 3.2. Ultrasonic-Assisted Extraction of Grapevine Roots Using NADES

**Figure 3:**
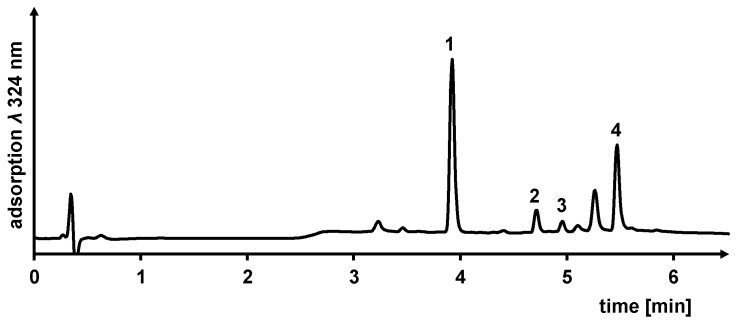
UHPLC-UV chromatogram monitored at *λ* 324 nm of a NADES grapevine root extract (Ch/Pdiol 1/2, 10 wt% H_2_O). (1), resveratrol *t_R_* 3.924 min; (2), ε-viniferin *t_R_* 4.645 min; (3), r-2-viniferin *t_R_* 4.955 min; (4) r-viniferin *t_R_* 5.468 min.

The suitability of ultrasonic-assisted NADES extraction of stilbenoids from grapevine plant material was evaluated in a previous study [35]. Based on these results, rapid ultrasonic-assisted NADES extraction of the grapevine roots was performed, and the extracted stilbenoids were quantified within 7.2 min using a solvent-saving UHPLC-UV analytical method. Figure 3 shows a UHPLC-UV chromatogram measured at *λ* 324 nm of the stilbenoids resveratrol (1), ε viniferin (2), r-2-viniferin (3), and r-viniferin (4) extracted from grapevine roots using a Ch/Pdiol based NADES (molar ratio 1/2, 10 wt% H_2_O). In addition to the four main stilbenoids, the Ch/Pdiol based NADES extract also contained six other stilbenoids, including ampelopsin A, hopeaphenol and miyabenol C, as identified by HPLC-ESI-MS/MS (electrospray ionization operated in negative ion mode). Please see Supplementary Material Table S6 for more information. This demonstrated that the Ch/Pdiol based grapevine root extract has equivalent selectivity in stilbenoid composition compared to commercially available grapevine root extracts, extracted using conventional solvents, e.g., ethanol/water mixture [19].

The NADES selected in Section 3.1 were used as extraction agents, yielding extraction levels of resveratrol ranging from 0.52–2.47 mg/g DW and ε-viniferin ranging from 0.07–0.78 mg/g DW. For r-2-viniferin, a tetrameric stilbenoid, the extraction content was between 0 and 1.10 mg/g DW. Of the four main stilbenoids, r-viniferin had the highest extraction content ranging between 0.44 and 7.25 mg/g DW (Table 2).

**Figure 4:**
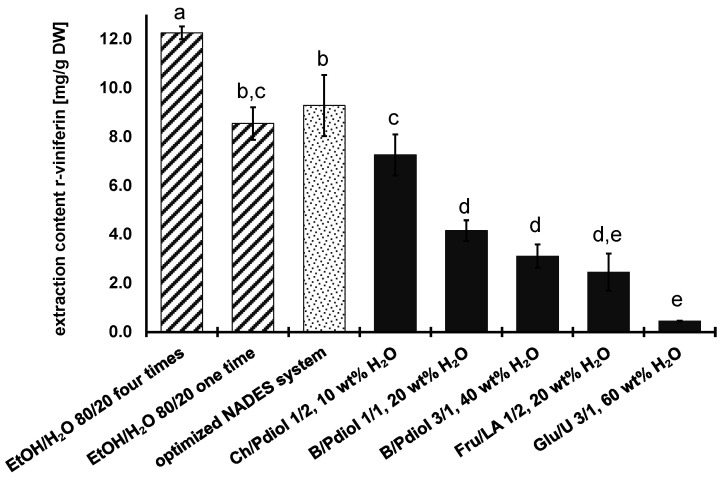
Extraction content of r-viniferin extracted from grapevine roots with ethanol/water (shaded) and different NADES systems (data are expressed as the mean value ± SD; means in the group with different letters (a–e) differ significantly at *p* < 0.05 as measured by Tukey’s HSD Test). Abbreviations: B, betaine; Ch, choline chloride; Fru, fructose; Glu, glucose; LA, lactic acid; Pdiol, 1,2-propanediol; and U, urea.

The Ch/Pdiol 1/2, 10 wt% H_2_O system was the most suitable NADES for the extraction of r-viniferin (7.25 ± 0.84 mg/g DW), which differed significantly from the other systems in extraction yield, as shown in Figure 4. With a NADES system consisting of B/Pdiol 1/1, 20 wt% H_2_O, the second highest extraction content of 4.16 ± 0.42 mg/g DW was achieved. The Glu/U 3/1, 60 wt% H_2_O system has the lowest extraction content for r-viniferin (0.44 ± 0.02 mg/g DW). The extraction efficiency of the other stilbenoids ε-viniferin, r-2-viniferin, and r-viniferin was best when the Ch/Pdiol 1/2, 10 wt% H_2_O system was used. The results demonstrate that NADES with polyols as HBDs extracted the highest levels of monomeric to tetrameric stilbenoids. This finding is consistent with the experimental work of Chen et al., (2018) reported for resveratrol [50]. Furthermore, these extraction experiments demonstrated that the capacity of the NADES to extract stilbenoids decreases with increasing water content in the systems. In the NADES Glu/U (3/1, 60 wt% H_2_O), the hydrogen-bonding interactions between the HBA and HBD weaken. This results in a solvent with an aqueous character that is less effective in extracting the stilbenoids [51].

When comparing the extraction contents of r-viniferin obtained through single-step extraction using Ch/Pdiol (7.25 ± 0.84 mg/g DW) and the ethanol/water mixture (8.54 ± 0.66 mg/g DW), a significantly higher amount of r-viniferin was extracted using the conventional mixture. During both single-step extractions, resveratrol, ε-viniferin, and r-2-viniferin were extracted almost completely. Further optimization of the extraction parameters is necessary because parameters such as the b/N ratio and extraction time could not be calculated in advance with the COSMO-RS, as is the case for the water content and molar ratio of the NADES systems. Therefore, in the optimization process of NADES extraction, the extraction parameters were optimized using r-viniferin as a reference.

The NADES extraction showed its potential as an alternative extractant, and compared to conventional organic solvent/water mixtures, it has the advantage of eliminating the solvent removal step. This enables new applications, such as the use of Ch/Pdiol or B/Pdiol based ready-to-use NADES grapevine root extracts, where these resulting ready-to-use extracts combine the bioactivities of antioxidative and tyrosinase inhibitory effects of a grapevine root extract and the skin-lightening effect of betaine [17,43]. Furthermore, a Fru/LA-based NADES extract was prepared with food-grade ingredients; therefore, it is suitable to be directly used in food products. To assess the applicability of the ready-to-use grapevine root extracts, the storage stability of the stilbenoids in these extracts had to be evaluated (cf. Section 3.4). In the following section, the NADES-based extraction process is optimized for the most promising system so far, consisting of Ch/Pdiol.

### 3.3. Optimization of the NADES-Based Extraction Process

The extraction process based on the NADES has some difficulties as the used components reduce mass transfer during extraction due to their much higher viscosities compared to organic solvents and water. Therefore, one important aim was to optimize the ultrasonic-assisted extraction process of grapevine roots with the optimal NADES Ch/Pdiol for the extraction of stilbenoids using r-viniferin as the representative target molecule. For all stilbenoids, data and statistical analysis (ANOVA, Tukey’s HSD test) are listed in Supplementary Material Table S7. The effect of parameters—including water content, molar ratio, biomass/NADES ratio (b/N ratio), and time—on the capacity of the NADES Ch/Pdiol to extract r-viniferin from grapevine roots were analyzed sequentially, and the optimal extraction conditions for each parameter were defined.

#### 3.3.1. Influence of Water Content in NADES on r-Viniferin Extraction Capacity

**Figure 5:**
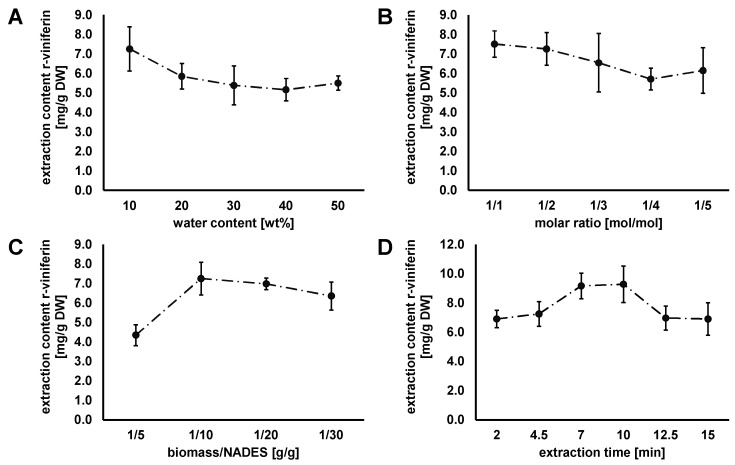
Effect of (**A**) water content (fixed Ch/Pdiol 1/2, 1/10 g/g b/N ratio, 4.5 min), (**B**) Ch/Pdiol molar ratio (fixed Ch/Pdiol, 10 wt% H_2_O, 1/10 g/g b/N ratio, 4.5 min), (**C**) biomass/NADES ratio (fixed Ch/Pdiol 1/2, 10 wt% H_2_O, 4.5 min), (**D**) extraction time (fixed Ch/Pdiol 1/2, 10 wt% H_2_O, 1/10 g/g b/N ratio) on the extraction content of r-viniferin from grapevine roots (data are expressed as the mean value ± SD).

The extraction capacity of the NADES systems is influenced by the water content since they have a higher viscosity compared to organic solvents. Since some molar ratios of Ch/Pdiol were not stable without the addition of water, the optimization was carried out with water contents in the range of 10–50 wt%. The remaining parameters were kept constant (molar ratio 1/2 mol/mol, b/N ratio 1/10 g/g, and extraction time 4.5 min). As presented in Figure 5A, the significantly highest yield of r-viniferin was achieved when 10 wt% water was added into the NADES system (*p* < 0.05). The extraction capacity of the NADES was observed to decrease with increasing water contents. This could be explained by a decrease in hydrogen-bond interaction between the HBA and HBD [51]. These observations were also made for the extraction levels of the other stilbenoids. The optimum amount of water was then chosen to be 10 wt%.

#### 3.3.2. Influence of the Choline Chloride/1,2-Propanediol Molar Ratio on r-Viniferin Extraction

The effect of the Ch/Pdiol molar ratio (1/1, 1/2, 1/3, 1/4, and 1/5 mol/mol) on the extraction content of r-viniferin was examined, with the following parameters fixed: water content, 10 wt% H_2_O; b/N ratio, 1/10 g/g; and extraction time, 4.5 min. The results presented in Figure 5B showed that the maximum extraction content of r-viniferin (7.50 ± 0.67 mg/g DW) could be achieved at the Ch/Pdiol molar ratio of 1/1, which also applies to ε-viniferin. In the case of resveratrol, the significantly highest extraction content is achieved with a molar ratio of 1/2 (*p* < 0.05). Therefore, the total stilbenoid content was considered for the selection of the appropriate molar ratio, which is highest at a ratio of 1/2. Therefore, the Ch/Pdiol molar ratio of 1/2 (mol/mol) was selected for the next parameter optimization experiments. Finally, it should be pointed out that the molar ratio of Ch/Pdiol has no significant influence (*p* < 0.05) on the extraction content of ε-viniferin, r-2-viniferin, and r-viniferin.

#### 3.3.3. Influence of the Biomass/NADES Ratio on r-Viniferin Extraction

The results showed that as the b/N ratio increased from 1/5 up to 1/10 g/g (as shown in Figure 5C), the extraction yield of r-viniferin also increased, while the molar ratio parameters (1/2 mol/mol), water content (10 wt% H_2_O), and extraction time (4.5 min) were kept constant. As the b/N ratio increases to 1/30, the extraction content of r-viniferin decreases. The same trend was observed for the other stilbenoids (Supplementary Material Table S7). The extraction efficiency decreased when the b/N ratio was too high because the extractants could not completely extract r-viniferin due to the viscosity-related low mass transfer of NADES [11]. An improvement in ultrasonic-assisted extraction with a larger extraction volume could be achieved by stirring. The b/n ratio of 1/10 was selected for the next experiment.

#### 3.3.4. Influence of the Extraction Time on r-Viniferin Extraction

In this experiment, the ultrasonic-assisted extraction time was optimized for the r-viniferin recovery under the above optimized conditions (1/2 mol/mol, 10 wt% H_2_O, 1/10 g/g b/N ratio). The extraction time was set to 2, 4.5, 7, 10, 12.5, and 15 min. When the extraction time was increased from 2 to 10 min, the extraction content of r-viniferin showed a significant increase (*p* < 0.05). However, with further extension of the extraction time, the extraction content of r-viniferin decreased again (cf. Figure 5D). These observations were also made for the extraction contents of the other stilbenoids. The extraction of r-viniferin was most effective at an extraction time of 10 min, yielding 9.28 ± 1.25 mg/g DW and 14.31 ± 1.60 mg/g DW for all stilbenoids. A longer extraction time may lead to polymerization reactions of the stilbenoids by high energy input, resulting in decreasing extraction contents.

In conclusion, the ultrasonic-assisted extraction process for resveratrol, ε-viniferin, r-2-viniferin, and r-viniferin from grapevine roots was optimized sequentially by using the Ch/Pdiol-based NADES. The following parameters were obtained: molar ratio 1/2 mol/mol, water content 10 wt%, b/N ratio 1/10 g/g, and extraction time 10 min. By optimizing the various extraction parameters, the extraction content was increased by 28% and 23% for r-viniferin and stilbenoids, respectively. Ultrasonic-assisted extraction using the NADES Ch/Pdiol with the optimum extraction conditions yielded a significant (*p* < 0.05) higher r-viniferin content compared with conventional single-cycle extraction using ethanol/water mixture (80/20; *v*/*v*) (cf. Figure 4). A comparison of the extraction contents of the other stilbenoids, which were obtained under optimized conditions, showed that they were already completely extracted via a single extraction step (cf. Table 2). Overall, 83% of the stilbenoids were extracted using the NADES Ch/Pdiol 1/2, 10 wt% H_2_O in a single extraction step under optimized conditions, compared to four times extraction with ethanol/water mixture (80/20; *v*/*v*). This indicates that ultrasonic-assisted stilbenoid extraction from grapevine roots using the NADES Ch/Pdiol is highly effective and a real process alternative.

### 3.4. Stability Study of Ready-to-Use NADES Extracts

It was essential to evaluate the stability of stilbenoids within the NADES extracts to determine their ability as ready-to-use formulations due to a lack of available experimental data. For this reason, the storage stability of the four main stilbenoids in different NADES systems was monitored. Therefore, only their extraction yields were determined, and degradation products were not quantified. For these stability studies, grapevine root extracts were prepared using the Ch/Pdiol and Fru/LA-based NADES systems. The Ch/Pdiol grapevine root extract could be applied in cosmetic formulations. Similar NADES compositions have been previously reported for extracting resveratrol from mulberry callus using Ch/Gly [38] and from grapevine canes or peanut roots using Ch/Bdiol [44,50]. The Fru/LA grapevine root extract was selected for the storage stability study because of its potential use in foods and the previously described ability of acidic NADES to extract resveratrol from *Gnetum gnemon* seeds (B/LA), *Polygonum cuspidatum* roots (Ch/OxA), *Morus alba* callus (Ch/MA), and grapevine shoots (Ch/levulinic acid) [10,11,38,45].

#### 3.4.1. Stability Study of Stilbenoids in Choline Chloride/1,2-Propanediol NADES

**Figure 6:**
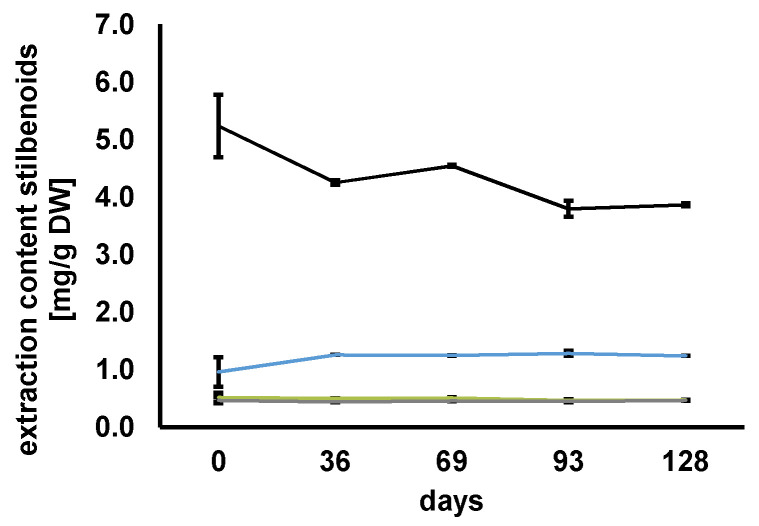
Monitoring the storage stability of resveratrol (blue), ε-viniferin (green), r-2-viniferin (gray), and r-viniferin (black) in the grapevine root ready-to-use NADES extract produced with Ch/Pdiol 1/2, 10 wt% H_2_O over 128 days.

The storage stability of the stilbenoids can be affected by UV radiation, temperature, and pH value of the NADES. The NADES grapevine root extracts were stored and protected from light at ambient temperature for 128 days. The pH values for Ch/Pdiol 1/2, 10 wt% H_2_O and Fru/LA 1/2, 20 wt% H_2_O systems were 6.03 and 1.33, respectively. The NADES Ch/Pdiol 1/2, 10 wt% H_2_O was shown to be appropriate for the stabilization of the stilbenoids resveratrol, ε-viniferin, and r-2-viniferin. Figure 6 shows no significant differences (*p* < 0.001) in the values of these stilbenoids within 128 days of storage (statistical analysis ANOVA and Tukey’s HSD test are listed in Supplementary Material Table S8). Over the first 36 days of storage, a minor decrease in the r-viniferin content was observed. Following this period, the r-viniferin content remained stable. This result allows the preparation of a Ch/Pdiol grapevine root ready-to-use extract containing 0.1% resveratrol, 0.05% ε-viniferin, 0.08% r-2-viniferin, and 0.45% r-viniferin (0.69% stilbenoids overall). The extract was found to be stable for 128 days when stored at ambient temperature (22 °C) in the dark. Further research on storage conditions using Ch/Pdiol grapevine root ready-to-use extract at lower temperatures or utilizing a NADES system with reduced water content could lead to further improvement of its storage stability [38].

#### 3.4.2. Stability Study of Stilbenoids in Fructose/Lactic Acid NADES

In a first stability study of the stilbenoids in Fru/LA grapevine root extract, it was observed that the stilbenoids were completely degraded within the first 36 days (data not shown). Therefore, the storage study of stilbenoids in the Fru/LA grapevine root extract has to be repeated using smaller analysis intervals. In addition, a model study could be performed using a Fru/LA-based NADES spiked with resveratrol.

**Figure 7:**
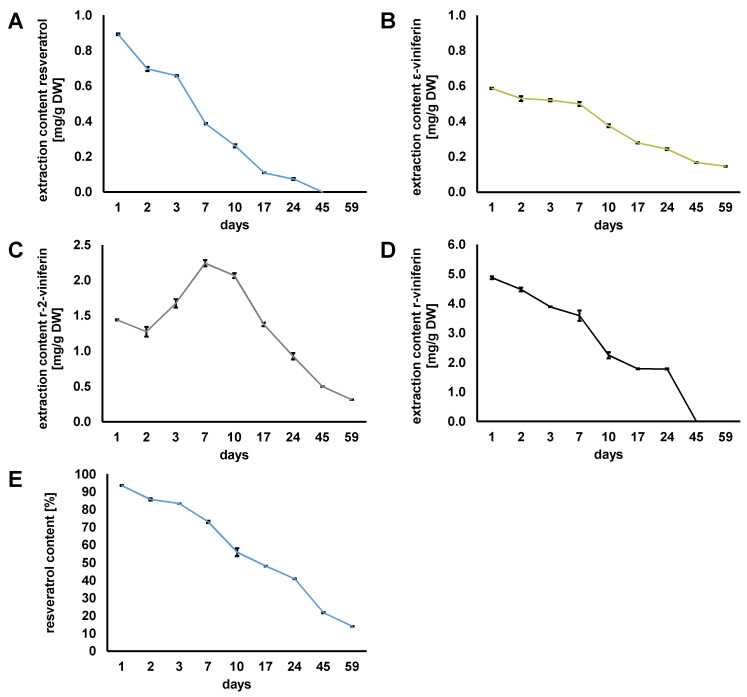
Monitoring the storage stability of (**A**) resveratrol, (**B**) ε-viniferin, (**C**) r-2-viniferin, (**D**) r-viniferin in the grapevine root ready-to-use NADES extract produced with Fru/LA 1/2, 20 wt% H_2_O over 59 days. The stability of resveratrol reference standard in the Fru/LA 1/2, 20 wt% H_2_O NADES system over 59 days was analyzed for comparison purposes (**E**).

When the storage stability of stilbenoids in the ready-to-use Fru/LA grapevine root extract was monitored in smaller analysis intervals, it was found that the content of resveratrol was significantly decreased (*p* < 0.05, statistical analysis ANOVA, Tukey’s HSD test are listed in Supplementary Material Table S9), which is no longer detectable after 45 days (Figure 7A). Figure 7B shows a significant decrease (*p* < 0.05) in ε-viniferin content in the NADES, indicating that the dimer was also not stable in Fru/LA. For r-2-viniferin, the trend was different (Figure 7C). There was a significant increase (*p* < 0.05) in the tetramer content within the initial seven days. After that, the r-2-viniferin content decreased steadily. The content of r-viniferin significantly decreased (*p* < 0.05) until day 24 and finally became undetectable after 45 days (Figure 7D). From the next measurement date, statistical analysis revealed no difference in r-viniferin content in the NADES after day 10 (*p* < 0.05).

Figure 7E shows a model study where the NADES Fru/LA was spiked with resveratrol; significant degradation of the stilbenoid was observed within 59 days (*p* < 0.05, statistical analysis ANOVA and Tukey’s HSD test are listed in Supplementary Material Table S10). The 85% degradation was confirmed by the observations of Komaikul et al. (2021), who reported decreasing resveratrol content in spiked the Ch/MA-based NADES [38]. In conclusion, it was found that the stilbenoids extracted from the grapevine roots were not stable during storage in the acidic Fru/LA NADES, similar to resveratrol in the model experiment.

**Figure 8:**
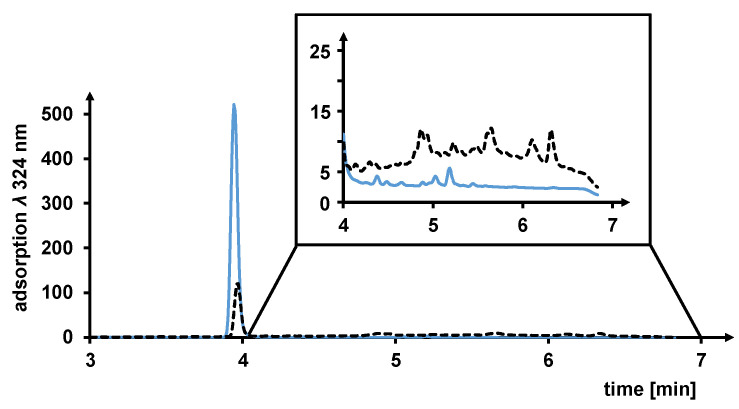
UHPLC-UV chromatogram monitored at *λ* 324 nm of resveratrol (*t_R_* 3.931 min) stored for 1 day (**–**) and 45 days (- -) in the NADES system consisting of Fru/LA 1/2, 20 wt% H_2_O). Expansion of the retention time range 4–7 min, where the oligomeric stilbenoids were eluted.

HPLC-ESI-MS/MS was used to investigate which compounds were formed during the degradation of resveratrol, but the identified main pseudo molecular ions [M−H]^−^ *m*/*z* 545 (fragment ions: *m*/*z* 527, 503, 487, 383, 339, 225) and [M−H]^−^ *m*/*z* 493 (fragment ions: *m*/*z* 465, 449, 343, 265, 237, 227, 209, 193) of the new substances did not agree with the common data for oligomers, which were generated by the plant’s own enzymatic processes [13]. Based on the increased UV absorption at *λ* 324 nm in the retention time range of 5–6 min and the acidity of the NADES, the hypothesis is that the oligomerization of resveratrol was catalyzed by lactic acid (Figure 8). A similar dimerization phenomenon of resveratrol was described using formic acid [52].

In accordance with the literature [10,11,38,45], it was shown that the acidic NADES composed of Fru/LA was able to extract the four stilbenoids. However, this stability study was the first to show that the stilbenoids investigated in the present study were degraded during storage in the acidic Fru/LA-based ready-to-use extract. Therefore, the removal of the NADES is necessary when acidic NADES are used. Conversely, the use of polyols as HBDs results in the storage of the stable NADES extracts, allowing for broader use, for example, as a ready-to-use extract.

### 3.5. Recovery of Extracted Stilbenoids

Compared to conventional organic solvents, the NADES have a low vapor pressure, which makes the removal of the NADES components from extracts impossible by low-vacuum distillation. However, several approaches, including liquid–liquid extraction, solid-liquid extraction, and the use of anti-solvents, can be used to recover the extracted target compounds from the NADES mixtures [53]. In this study, the stilbenoids were recovered from the NADES extract using the macroporous adsorbent resin Amberlite^®^ XAD-16N. The NADES extract from grapevine roots was prepared as described in Section 2.5, using the NADES system consisting of Ch/Pdiol 1/2, 10 wt% H_2_O. The extract was passed through a column filled with the XAD-16N resin, and the NADES components were removed by washing with deionized water, and the adsorbed stilbenoids were subsequently eluted by ethanol. The applied strategy to remove the NADES components from stilbenoids was highly efficient, achieving yields of 87%, 84%, 84%, and 80% for resveratrol, ε-viniferin, r-2-viniferin, and r-viniferin, respectively. Finally, the stilbenoid content in the NADES-free extracts was 26.5% (2.4% resveratrol, 1.9% ε-viniferin, 1.7% r-2-viniferin, and 20.5% r-viniferin). Therefore, the process development for the recovery of stilbenoids from the NADES extracts resulted in a highly enriched extract of target molecules.

## 4. Conclusions

In summary, this study demonstrated for the first time a sustainable COSMO-RS-supported workflow for the ultrasonic-assisted extraction of resveratrol, ε-viniferin, r-2-viniferin, and r-viniferin from grapevine roots using the NADES as a green solvent. By screening potential HBA/HBD combinations for the stilbenoid extraction supported by the COSMO-RS, a suitable NADES consisting of choline chloride/1,2-propanediol was found. After a successful optimization of the extraction parameters (molar ratio 1/2 mol/mol, water content 10 wt%, biomass/NADES ratio 1/10 g/g, and extraction time 10 min), an extraction yield of r-viniferin of 9.28 ± 1.25 mg/g DW was achieved, which is significantly higher than using a conventional ethanol/water mixture (80/20; *v*/*v*) for extraction. These obtained ready-to-use NADES grapevine root extracts contained 0.69% stilbenoids. A NADES-based extraction offers an advantage over traditional ethanol/water extraction by eliminating the solvent removal step. Storage studies for ready-to-use extracts containing stilbenoids are still needed. Therefore, the choline chloride/1,2-propanediol grapevine root extract was stored for 128 days in order to evaluate its stability. No significant changes were observed in the extraction contents of stilbenoids, confirming the use of choline chloride/1,2-propanediol to produce ready-to-use grapevine root extracts for cosmetic formulations. When the stilbenoids were stored in the NADES consisting of fructose and lactic acid, degradation of the stilbenoids within 59 days was observed for the first time. Therefore, removing the NADES would be beneficial. Subsequently, the use of the XAD-16N resin demonstrated a viable approach to removing the NADES from the stilbenoid-containing extracts for potential applications as nutraceuticals in the food industry. After the removal of the NADES components, an extract enriched with stilbenoids was obtained, which contained 2.4% resveratrol, 1.9% ε-viniferin, 1.7% r-2-viniferin, and 20.5% r-viniferin. Overall, this study demonstrated a sustainable workflow tailored to stilbenoids for their extraction from side-streams of wine production using the environmentally friendly NADES, which could be transferred to the extraction of other natural compounds to become even more sustainable by exploiting other side-streams in the food industry.

## Figures and Tables

**Table 1:** Various compositions of the prepared NADES.

Abbreviation	Component 1 (HBA)	Component 2 (HBD)	Molar Ratio	Water Content (wt%)
Ch/Pdiol	Choline chloride	1,2-Propanediol	1/2	10
B/Pdiol	Betaine	1,2-Propanediol	1/1	20
B/Pdiol	Betaine	1,2-Propanediol	3/1	40
Fru/LA	Fructose	Lactic acid	1/2	20
Glu/U	Glucose	Urea	3/1	60

**Table 2:** Extraction contents of resveratrol, ε-viniferin, r-2-viniferin, and r-viniferin in different extractants. Abbreviations: B, betaine; Ch, choline chloride; Fru, fructose; Glu, glucose; LA, lactic acid; Pdiol, 1,2-propanediol; and U, urea. Data are expressed as the mean ± SD (milligrams per gram of dry weight) (*n* = 3); means in the group with different letters (a–f) differ significantly at *p* < 0.05 as measured by Tukey’s HSD Test.

Extractant	Resveratrol	ε-Viniferin	r-2-Viniferin	r-Viniferin
EtOH/H_2_O four times	2.41 ± 0.03 ^a,b^	1.01 ± 0.06 ^a^	1.49 ± 0.05 ^a,b^	12.25 ± 0.26 ^a^
EtOH/H_2_O one time	2.49 ± 0.09 ^a^	0.97 ± 0.05 ^a^	1.14 ± 0.08 ^a,b,c^	8.54 ± 0.66 ^b,c^
optimized NADES system *	2.47 ± 0.05 ^a^	0.95 ± 0.03 ^a^	1.61 ± 0.27 ^a^	9.28 ± 1.25 ^b^
Ch/Pdiol 1/2, 10 wt% H_2_O	2.47 ± 0.11 ^a^	0.78 ± 0.08 ^a,b^	1.10 ± 0.11 ^b,c,d^	7.25 ± 0.84 ^c^
B/Pdiol 1/1, 20 wt% H_2_O	2.20 ± 0.30 ^a,b^	0.70 ± 0.13 ^b,c^	0.81 ± 0.15 ^c,d,e^	4.16 ± 0.42 ^d^
B/Pdiol 3/1, 40 wt% H_2_O	1.87 ± 0.05 ^b^	0.48 ± 0.07 ^c,d^	0.47 ± 0.17 ^e,f^	3.11 ± 0.48 ^d^
Fru/LA 1/2, 20 wt% H_2_O	1.13 ± 0.37 ^c^	0.42 ± 0.12 ^d^	0.65 ± 0.27 ^d,e^	2.45 ± 0.76 ^d,e^
Glu/U 3/1, 60 wt% H_2_O	0.52 ± 0.05 ^d^	0.07 ± 0.01 ^e^	0.00 ± 0.00 ^f^	0.44 ± 0.02 ^e^

* Ch/Pdiol 1/2, 10 wt% H_2_O, 1/10 g/g biomass/NADES ratio, 10 min extraction time.

## Data Availability

The original contributions presented in the study are included in the article/Supplementary Material, further inquiries can be directed to the corresponding author.

## Supplementary Materials

The following supporting information can be downloaded at: https://www.mdpi.com/article/10.3390/foods13020324/s1, Figure S1: Various sigma profiles obtained by COSMOthermX of resveratrol, ε-viniferin, r-2-viniferin, and r-viniferin; Figure S2: COSMO charge density surfaces of resveratrol, ε-viniferin, r-2-viniferin, and r-viniferin; Figure S3: Sigma profiles obtained by COSMOthermX of choline chloride, 1,2-propanediol, water, and NADES system choline chloride/1,2-propanediol 1/2, 10 wt% H_2_O; Figure S4: COSMO charge density surfaces of choline chloride, 1,2-propanediol, and water; Figure S5: Heat map of the activity coefficients ln *γ* calculated with the COSMO-RS (25 °C) of (A) resveratrol, (B) ε-viniferin, and (C) r-2-viniferin in different NADES; Table S1: Activity coefficients ln *γ* calculated with the COSMO-RS (25 °C) of resveratrol in different NADES; Table S2: Activity coefficients ln *γ* calculated with the COSMO-RS (25 °C) of ε-viniferin in different NADES; Table S3: Activity coefficients ln *γ* calculated with the COSMO-RS (25 °C) of r-2-viniferin in different NADES; Table S4: Activity coefficients ln *γ* calculated with the COSMO-RS (25 °C) of r-viniferin in different NADES; Figure S6: Comparison of the COSMO-RS-calculated ln *γ* values and measured extraction contents of (A) resveratrol, (B) ε-viniferin, (C) r-2-viniferin, and (D) r-viniferin; Table S5: Quantification parameters of the UHPLC-UV methodology; Table S6: HPLC-ESI-MS/MS (negative operation mode) data of compounds identified in the grapevine root NADES extract (Ch/Pdiol 1/2, 10 wt% H_2_O); Table S7: Extraction contents of resveratrol, ε-viniferin, r-2-viniferin, and r-viniferin during the optimization process; Table S8: Extraction contents of resveratrol, ε-viniferin, r-2-viniferin, and r-viniferin in Ch/Pdiol 1/2, 10 wt% H_2_O NADES after different days of storage; Table S9: Extraction contents of resveratrol, ε-viniferin, r-2-viniferin, and r-viniferin in Fru/LA 1/2, 20 wt% H_2_O NADES after different days of storage; Table S10: Resveratrol contents in Fru/LA 1/2, 20 wt% H_2_O NADES after different days of storage.
