# Supplementary material for: Sustainable In Silico-Supported Ultrasonic-Assisted Extraction of Oligomeric Stilbenoids from Grapevine Roots Using Natural Deep Eutectic Solvents (NADES) and Stability Study of Potential Ready-to-Use Extracts
Source: Foods. 2024 Jan 19;13(2):324. doi: 10.3390/foods13020324 (PMC10815275; doi:10.3390/foods13020324)
Supplement: Supplementary file 1 [file foods-13-00324-s001.zip › foods-2823206-supplementary.pdf]

Supplementary Material

Sustainable In Silico-Supported Ultrasonic-Assisted Extraction of Oligomeric Stilbenoids from Grapevine Roots Using Natural Deep Eutectic Solvents (NADES) and Stability Study of Potential Ready-to-Use Extracts

Mats Kiene [1], Malte Zaremba [1], Edwin Januschewski [1,2], Andreas Juadjur [2], Gerold Jerz [1] and Peter Winterhalter [1,*]

[1] Institute of Food Chemistry, Technische Universität Braunschweig, Schleinitzstraße 20, 38106 Braunschweig, Germany; m.kiene@tu-braunschweig.de (M.K.); g.jerz@tu-braunschweig.de (G.J.)

[2] German Institute of Food Technologies, Chemical Analytics, Prof.-von-Klitzing-Straße 7, 49610 Quakenbrück, Germany; a.juadjur@dil-ev.de

* Correspondence: p.winterhalter@tu-braunschweig.de

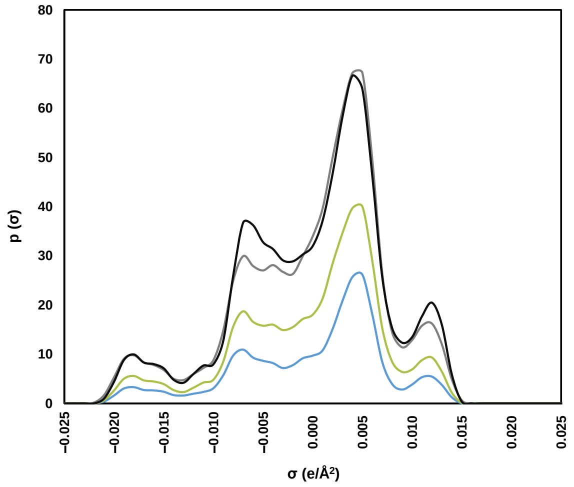

Figure S1. Various sigma profiles obtained by COSMOthermX of resveratrol (blue), ε-viniferin (green), r-2-viniferin (gray), and r-viniferin (black).

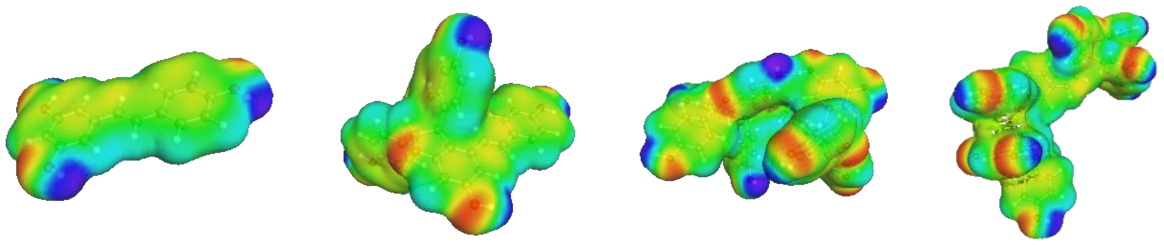

Figure S2. COSMO charge density surfaces of resveratrol, ε-viniferin, r-2-viniferin, and r-viniferin.

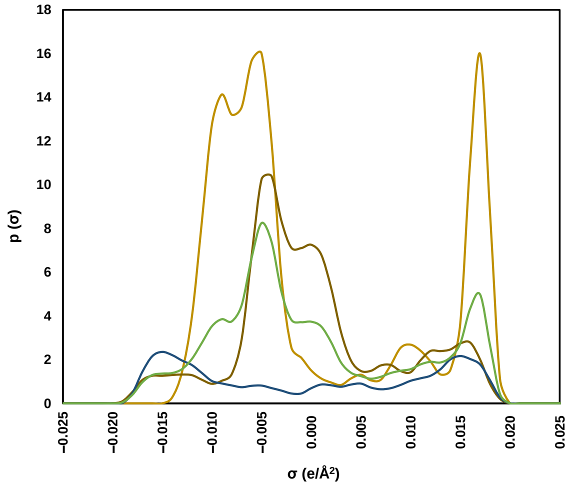

Figure S3. Sigma profiles obtained by COSMOthermX of choline chloride (orange), 1,2-propanediol (light brown), water (blue), and NADES system choline chloride/ 1,2-propanediol 1/2, 10 wt% H_2O (green).

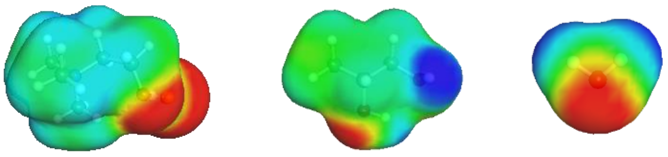

Figure S4. COSMO charge density surfaces of choline chloride, 1,2-propanediol, and water.

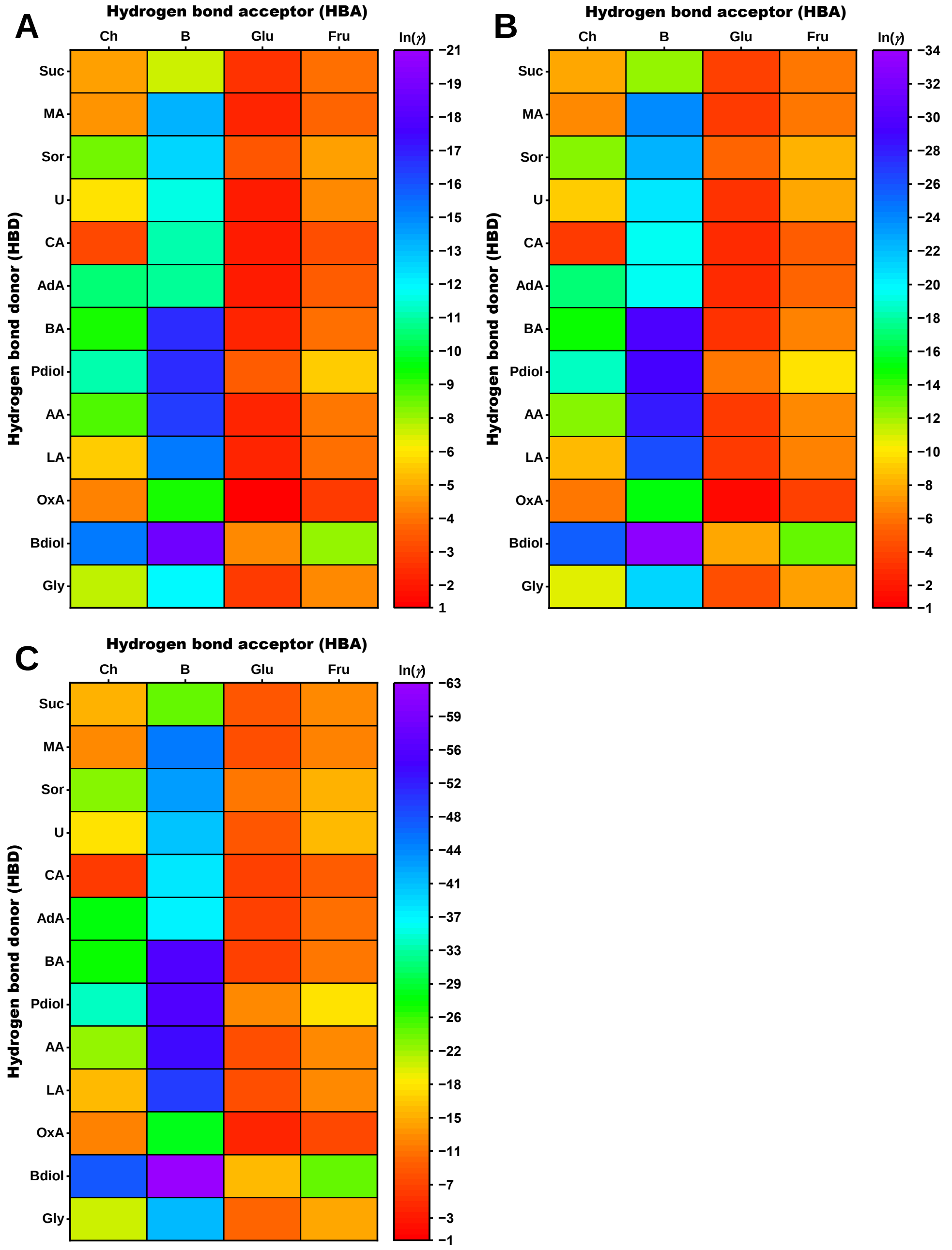

Figure S5. Heat map of the activity coefficients ln *γ* calculated with COSMO-RS (25 °C) of (A) resveratrol, (B) ε-viniferin, and (C) r-2-viniferin in different NADES. The molar ratio is 1/1 and the water content for all NADES is 0 wt%. Abbreviations: AA, acetic acid; AdA, adipic acid; BA, benzoic acid; B, betaine; Bdiol, 1,4-butanediol; Ch, choline chloride; CA, citric acid; Fru, fructose; Glu, glucose; Gly, glycerol; LA, lactic acid; MA, malic acid; OxA, oxalic acid; Pdiol, 1,2-propanediol; Sor, sorbitol; Suc, sucrose; U, urea.

Table S1. Activity coefficients ln *γ* calculated with COSMO-RS (25 °C) of resveratrol in different NADES. The molar ratio is 1/1 and the water content for all NADES is 0 wt%. Abbreviations: AA, acetic acid; AdA, adipic acid; BA, benzoic acid; B, betaine; Bdiol, 1,4-butanediol; Ch, choline chloride; CA, citric acid; Fru, fructose; Glu, glucose; Gly, glycerol; LA, lactic acid; MA, malic acid; OxA, oxalic acid; Pdiol, 1,2-propanediol; Sor, sorbitol; Suc, sucrose; U, urea.

**HBDs**	**HBAs**			
	**Ch**	**B**	**Glu**	**Fru**
Scu	–4.9020725	–7.06341768	–2.31637636	–3.8239671
MA	–4.74040815	–13.6751199	–1.99014682	–3.74822226
Sor	–7.92223007	–12.9079415	–3.21761108	–4.85838165
U	–6.25765924	–11.8204644	–1.72016955	–4.53848815
CA	–2.8528458	–11.1845354	–1.65862619	–3.17076817
AdA	–10.495892	–10.9664663	–1.73601523	–3.46048516
BA	–8.9996872	–16.676928	–1.90776139	–3.89666887
Pdiol	–11.203665	–16.677172	–3.38618485	–5.77922886
AA	–8.4708809	–16.2179418	–1.87966044	–4.05345277
LA	–5.78488566	–14.9683053	–1.91658625	–3.89193502
OxA	–4.17392688	–8.94038304	–0.60857735	–2.43936742
Bdiol	–14.8703285	–19.0348935	–4.53943578	–7.58030984
Gly	–7.17578482	–12.1318439	–2.55280565	–4.481936

Table S2. Activity coefficients ln *γ* calculated with COSMO-RS (25 °C) of ε-viniferin in different NADES. The molar ratio is 1/1 and the water content for all NADES is 0 wt%. Abbreviations: AA, acetic acid; AdA, adipic acid; BA, benzoic acid; B, betaine; Bdiol, 1,4-butanediol; Ch, choline chloride; CA, citric acid; Fru, fructose; Glu, glucose; Gly, glycerol; LA, lactic acid; MA, malic acid; OxA, oxalic acid; Pdiol, 1,2-propanediol; Sor, sorbitol; Suc, sucrose; U, urea.

**HBDs**	**HBAs**			
	**Ch**	**B**	**Glu**	**Fru**
Scu	–7.83314595	–12.2493867	–4.0191355	–6.45400722
MA	–6.94318983	–23.8255807	–3.58595658	–6.27132811
Sor	–12.7272501	–22.6737539	–5.60926765	–8.20432838
U	–9.2806325	–20.4518667	–3.47663668	–7.9298914
CA	–3.73364775	–19.5803421	–2.92104906	–5.19972863
AdA	–17.1431765	–19.5079653	–3.08712964	–5.75271791
BA	–14.9606313	–29.4144698	–3.43837313	–6.46381106
Pdiol	–18.5303389	–29.2826391	–6.16321327	–10.0485621
AA	–12.7517103	–28.300758	–3.55575517	–6.90590087
LA	–8.74668687	–26.1900144	–3.60911375	–6.63789424
OxA	–6.42497322	–15.1887309	–1.42494282	–4.08823139
Bdiol	–25.4939992	–33.3344283	–8.07947434	–13.216823
Gly	–11.0819306	–21.199588	–4.67594618	–7.67647994

Table S3. Activity coefficients ln *γ* calculated with COSMO-RS (25 °C) of r-2-viniferin in different NADES. The molar ratio is 1/1 and the water content for all NADES is 0 wt%. Abbreviations: AA, acetic acid; AdA, adipic acid; BA, benzoic acid; B, betaine; Bdiol, 1,4-butanediol; Ch, choline chloride; CA, citric acid; Fru, fructose; Glu, glucose; Gly, glycerol; LA, lactic acid; MA, malic acid; OxA, oxalic acid; Pdiol, 1,2-propanediol; Sor, sorbitol; Suc, sucrose; U, urea.

**HBDs**	**HBAs**			
	**Ch**	**B**	**Glu**	**Fru**
Scu	–14.8276922	–24.0795749	–8.72722518	–12.4456975
MA	–12.5408172	–45.1684827	–8.05333033	–11.5428057
Sor	–22.6400959	–43.0420126	–10.8642463	–14.6171343
U	–18.2309332	–40.2186829	–8.44937	–15.2271394
CA	–6.18487969	–37.5741287	–6.6860048	–9.5042266
AdA	–28.113725	–37.1960447	–6.62594406	–10.1866696
BA	–26.9570446	–55.1299865	–6.98433542	–10.9232217
Pdiol	–34.2085756	–55.3879061	–12.1801361	–18.1996727
AA	–22.2496636	–53.4715413	–7.84467298	–12.3747812
LA	–15.6486184	–49.6670453	–8.04764402	–12.0889504
OxA	–11.8842648	–28.2662194	–4.35451122	–7.47637232
Bdiol	–47.7381715	–62.5390554	–15.5293309	–24.1253429
Gly	–20.354264	–40.7184084	–9.77601726	–14.0592871

Table S4. Activity coefficients ln *γ* calculated with COSMO-RS (25 °C) of r-viniferin in different NADES. The molar ratio is 1/1 and the water content for all NADES is 0 wt%. Abbreviations: AA, acetic acid; AdA, adipic acid; BA, benzoic acid; B, betaine; Bdiol, 1,4-butanediol; Ch, choline chloride; CA, citric acid; Fru, fructose; Glu, glucose; Gly, glycerol; LA, lactic acid; MA, malic acid; OxA, oxalic acid; Pdiol, 1,2-propanediol; Sor, sorbitol; Suc, sucrose; U, urea.

**HBDs**	**HBAs**			
	**Ch**	**B**	**Glu**	**Fru**
Scu	–12.2142929	–20.4874477	–7.045484	–10.548838
MA	–9.73520253	–40.5058568	–6.81516251	–10.0933149
Sor	–19.5661532	–38.4120091	–9.26580226	–12.8514011
U	–14.2745354	–35.1398217	–7.37453273	–13.6762527
CA	–3.87168136	–33.1411305	–5.61355628	–8.19069236
AdA	–24.9289406	–32.4838208	–5.5241816	–8.78773096
BA	–23.5377778	–50.0996249	–6.05692509	–9.62738704
Pdiol	–30.0716511	–50.3056629	–10.6483683	–16.4087197
AA	–18.2265686	–48.3722443	–6.86044881	–11.0663722
LA	–12.5695809	–44.7833741	–7.00154431	–10.7387853
OxA	–10.2430971	–25.3936185	–4.39696091	–7.14321783
Bdiol	–42.8408713	–57.3724579	–13.4406685	–21.6544063
Gly	–17.1364442	–36.0607442	–8.65398033	–12.7099653

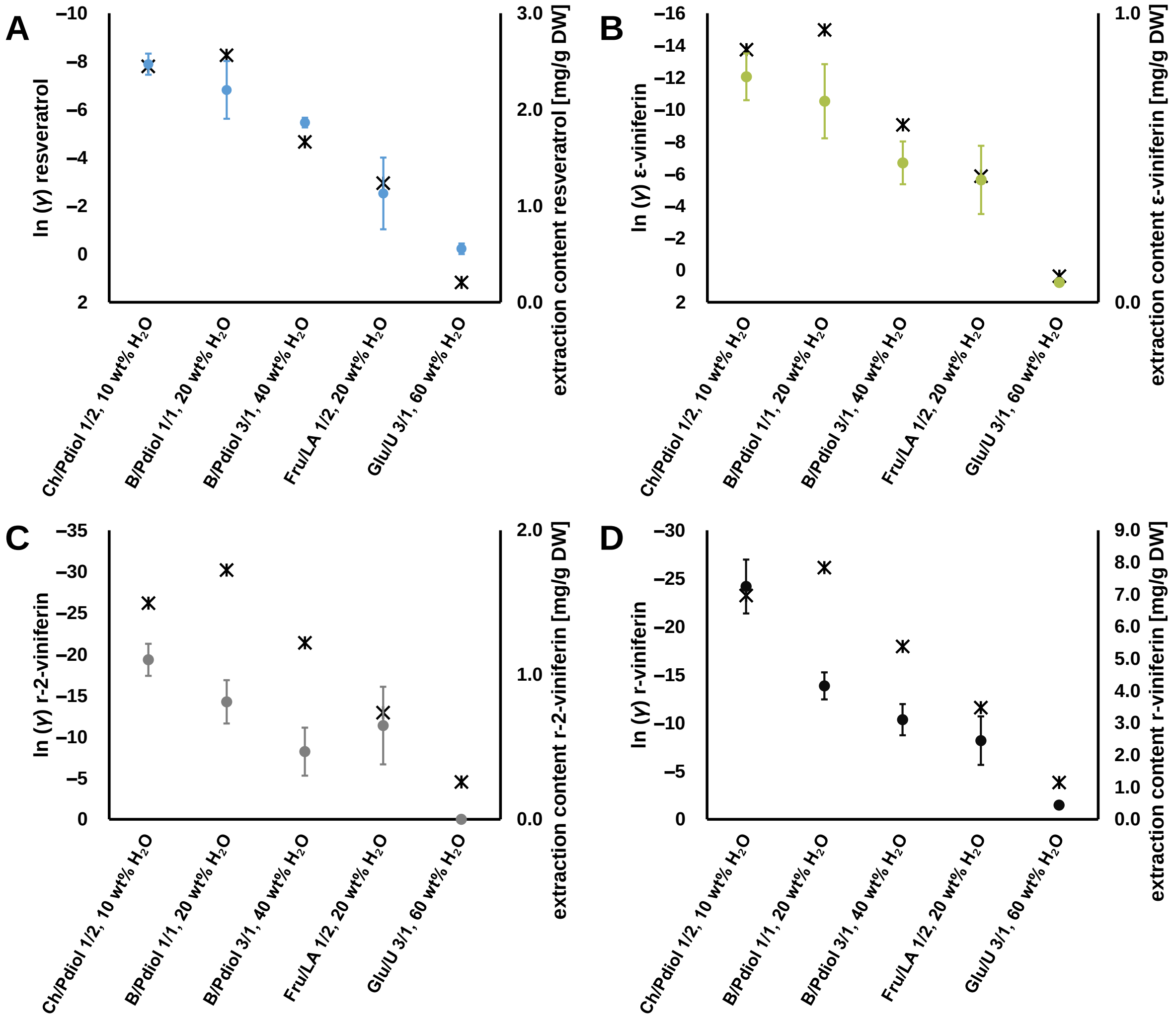

Figure S6. Comparison of COSMO-RS-calculated ln *γ* values (⁎) and measured extraction contents (●) of (A) resveratrol, (B) ε-viniferin, (C) r-2-viniferin, and (D) r-viniferin. Abbreviations: B, betaine; Ch, choline chloride; Fru, fructose; Glu, glucose; LA, lactic acid; Pdiol, 1,2-propanediol; U, urea.

Table S5. Quantification parameters of the UHPLC-UV methodology.

	**Resveratrol**	**ε-Viniferin**	**r-2-Viniferin**	**r-Viniferin**
Working range (mg/L)	2.5–20	2.5–20	0.4–43	12–120
R^2	0.9996	0.9993	0.9998	0.9986
t_R (min)	3.92	4.68	4.96	5.47
Limit of detection (mg/L)	0.43	0.61	0.14	0.43
Limit of quantification (mg/L)	1.50	2.10	0.55	1.53

Table S6. HPLC-ESI-MS/MS (negative operation mode) data of compounds identified in grapevine root NADES extract (Ch/Pdiol 1/2, 10 wt% H_2O).

**Compound**	t_R **[min]**	**Pseudo molecular ion [M–H]⁻ *m/z***	**Fragment ions *m/z***
Ampelopsin A	9.4	469	451, 375, 363
trans-Resveratrol	17.1	227	209, 185, 159, 143
Hopeaphenol	22.4	905	811, 717, 611, 451, 359
tetrameric Stilbenoid	24.2	905	811, 717, 707, 611, 451, 357
trans-ε-Viniferin	27.2	453	435, 411, 385, 359, 347, 289, 253, 225
Miyabenol C	30.0	679	661, 673, 585, 573, 479, 451, 345
r-2-Viniferin	31.0	905	887, 811, 705, 675, 545, 451, 359
trimeric Stilbenoid	34.7	679	673, 585, 447, 491, 357
r-Viniferin	35.8	905	887, 799, 705, 675, 545, 451, 359
tetrameric Stilbenoid	38.2	905	887, 811, 799, 705, 545, 451, 359

Table S7. Extraction contents of resveratrol, ε-viniferin, r-2-viniferin, and r-viniferin during the optimization process. Data are expressed as the mean ± SD [milligrams per gram of dry weight] (n = 3); means in the group with different letters (a-c) differ significantly at $p < 0.05$ as measured by Tukey's HSD Test.

		**Resveratrol**	**ε-Viniferin**	**r-2-Viniferin**	**r-Viniferin**
**Water content** [1]	10	2.47 ± 0.11^{a}	0.78 ± 0.08^{a}	1.10 ± 0.11^{a}	11.61 ± 1.14^{a}
[wt% H_2O]	20	2.57 ± 0.08^{a}	0.83 ± 0.06^{a}	0.78 ± 0.08^{b}	10.02 ± 0.66^{a,b}
	30	2.39 ± 0.10^{a}	0.77 ± 0.09^{a}	0.68 ± 0.12^{b}	9.22 ± 1.00^{b}
	40	1.93 ± 0.07^{b}	0.70 ± 0.09^{a}	0.66 ± 0.08^{b}	8.44 ± 0.58^{b}
	50	1.89 ± 0.10^{b}	0.69 ± 0.04^{a}	0.75 ± 0.02^{b}	8.84 ± 0.37^{b}
**HBA/HBD molar ratio** [2]	1/1	1.95 ± 0.01^{b,c}	0.81 ± 0.07^{a}	1.06 ± 0.12^{a}	7.50 ± 0.67^{a}
[mol/mol]	1/2	2.47 ± 0.11^{a}	0.78 ± 0.08^{a}	1.10 ± 0.11^{a}	7.25 ± 0.84^{a}
	1/3	2.31 ± 0.08^{a,b}	0.75 ± 0.10^{a}	0.87 ± 0.24^{a}	6.54 ± 1.50^{a}
	1/4	1.76 ± 0.28^{c}	0.66 ± 0.02^{a}	0.75 ± 0.10^{a}	5.71 ± 0.56^{a}
	1/5	1.61 ± 0.18^{c}	0.61 ± 0.10^{a}	0.79 ± 0.19^{a}	6.14 ± 1.16^{a}
**Biomass/NADES ratio** [3]	1/5	1.47 ± 0.09^{c}	0.46 ± 0.04^{b}	0.57 ± 0.06^{c}	4.35 ± 0.53^{b}
[g/g]	1/10	2.47 ± 0.11^{a}	0.78 ± 0.08^{a}	1.10 ± 0.11^{a}	7.25 ± 0.84^{a}
	1/20	2.18 ± 0.05^{b}	0.82 ± 0.05^{a}	0.95 ± 0.06^{a,b}	6.98 ± 0.30^{a}
	1/30	2.10 ± 0.03^{b}	0.77 ± 0.08^{a}	0.84 ± 0.11^{b}	6.36 ± 0.72^{a}
**Extraction time** [4]	2	1.80 ± 0.08^{b}	0.78 ± 0.09^{a}	0.80 ± 0.08^{c}	6.90 ± 0.59^{b}
[min]	4.5	2.47 ± 0.10^{a}	0.78 ± 0.08^{a}	1.10 ± 0.11^{b,c}	7.25 ± 0.84^{b}
	7	2.36 ± 0.01^{a}	0.92 ± 0.09^{a}	1.44 ± 0.14^{a,b}	9.17 ± 0.88^{a}
	10	2.47 ± 0.05^{a}	0.95 ± 0.03^{a}	1.61 ± 0.27^{a}	9.28 ± 1.25^{a}
	12.5	2.49 ± 0.13^{a}	0.79 ± 0.07^{a}	1.23 ± 0.20^{a,b,c}	6.97 ± 0.82^{b}
	15	2.54 ± 0.05^{a}	0.95 ± 0.09^{a}	1.43 ± 0.20^{a,b}	6.90 ± 1.11^{b}

[1] fixed Ch/Pdiol, 1/2 mol/mol, 1/10 g/g b/N ratio, 4.5 min
[2] fixed Ch/Pdiol, 10 wt% H_2O, 1/10 g/g b/N ratio, 4.5 min
[3] fixed Ch/Pdiol, 1/2 mol/mol, 10 wt% H_2O, 4.5 min
[4] fixed Ch/Pdiol, 1/2 mol/mol, 10 wt% H_2O, 1/10 g/g b/N ratio

Table S8. Extraction contents of resveratrol, ε-viniferin, r-2-viniferin, and r-viniferin in Ch/Pdiol 1/2, 10 wt% H_2O NADES after different days of storage. Data are expressed as the mean ± SD [milligrams per gram of dry weight] (n = 4), means in the group with different letters (a-c) differ significantly at $p < 0.001$ as measured by Tukey's HSD Test.

**Days**	**Resveratrol**	**ε-Viniferin**	**r-2-Viniferin**	**r-Viniferin**
0	0.961 ± 0.255^{a}	0.521 ± 0.081^{a}	0.471 ± 0.057^{a}	5.234 ± 0.54^{a}
36	1.255 ± 0.001^{a}	0.502 ± 0.002^{a}	0.441 ± 0.008^{a}	4.254 ± 0.039^{b,c}
69	1.244 ± 0.001^{a}	0.515 ± 0.004^{a}	0.462 ± 0.006^{a}	4.543 ± 0.018^{b}
93	1.284 ± 0.045^{a}	0.474 ± 0.016^{a}	0.453 ± 0.015^{a}	3.795 ± 0.143^{c}
128	1.241 ± 0.003^{a}	0.476 ± 0.001^{a}	0.465 ± 0.002^{a}	3.864 ± 0.024^{c}

Table S9. Extraction contents of resveratrol, ε-viniferin, r-2-viniferin, and r-viniferin in Fru/LA 1/2, 20 wt% H_2O NADES after different days of storage. Data are expressed as the mean ± SD [milligrams per gram of dry weight] (n = 4), means in the group with different letters (a-h) differ significantly at $p < 0.05$ as measured by Tukey's HSD Test.

Days	Resveratrol	ε-Viniferin	r-2-Viniferin	r-Viniferin
1	0.893 ± 0.005^{a}	0.587 ± 0.004^{a}	1.441 ± 0.009^{d}	4.877 ± 0.051^{a}
2	0.697 ± 0.012^{b}	0.529 ± 0.013^{b}	1.274 ± 0.069^{e}	4.478 ± 0.065^{b}
3	0.658 ± 0.004^{c}	0.520 ± 0.006^{b}	1.675 ± 0.060^{c}	3.889 ± 0.011^{c}
7	0.387 ± 0.004^{d}	0.499 ± 0.012^{c}	2.242 ± 0.042^{a}	3.589 ± 0.176^{d}
10	0.262 ± 0.008^{e}	0.376 ± 0.008^{d}	2.066 ± 0.035^{b}	2.245 ± 0.103^{e}
17	0.108 ± 0.002^{f}	0.279 ± 0.003^{e}	1.378 ± 0.025^{d}	1.784 ± 0.015^{f}
24	0.074 ± 0.006^{g}	0.243 ± 0.005^{f}	0.927 ± 0.046^{f}	1.778 ± 0.019^{f}
45	n.d.	0.168 ± 0.002^{g}	0.498 ± 0.004^{g}	n.d
59	n.d.	0.146 ± 0.002^{h}	0.313 ± 0.004^{h}	n.d.

n.d., not detected

Table S10. Resveratrol contents in Fru/LA 1/2, 20 wt% H_2O NADES after different days of storage (start content of resveratrol: 2.08 mg/g NADES). Data are expressed as the mean ± SD [milligrams per gram of NADES] (n = 4), means in the group with different letters (a-i) differ significantly at $p < 0.05$ as measured by Tukey's HSD Test.

Days	Resveratrol
1	1.878 ± 0.005^{a}
2	1.719 ± 0.012^{b}
3	1.673 ± 0.001^{c}
7	1.465 ± 0.013^{d}
10	1.122 ± 0.044^{e}
17	0.965 ± 0.003^{f}
24	0.821 ± 0.003^{g}
45	0.436 ± 0.006^{h}
59	0.281 ± 0.002^{i}
